# Prenatal detection and molecular cytogenetic characterization of Xp deletion and Xq duplication: a case report and literature review

**DOI:** 10.1186/s12920-024-01824-8

**Published:** 2024-02-21

**Authors:** Qing Lin, Chunya Liang, Bole Du, Lijiao Li, Hong Li, Xiaolan Mai, Sheng Li, Wenyu Xu, Cunzhen Wu, Mi Zeng

**Affiliations:** 1 Center of Prenatal Diagnosis, Zhanjiang Maternity and Child Health Care Hospital, Zhanjiang, China; 2 Guangzhou Jingke Biotechnology Co., Ltd, Guangzhou, P. R. China

**Keywords:** Noninvasive prenatal screening, Prenatal diagnosis, Chromosome microarray analysis, Xp22.33p22.32 deletion, Xq27.1q28 duplication

## Abstract

**Background:**

Copy number variation (CNV) of X chromosome can lead to a variety of neonatal abnormalities, especially for male fetuses. In recent years, due to the high sensitivity and high specificity of NIPS, its application has gradually expanded from chromosome aneuploidy to CNV. Few prenatal cases involving the detection of Xq duplication and deletion by NIPS have been reported, but it is of great significance for genetic counseling.

**Case presentation:**

A 36-year-old woman was referred for prenatal diagnosis and genetic counseling at 17 weeks of gestation because of abnormal result of noninvasive prenatal screening (NIPS). Multiple congenital malformations, hydrocephalus, and enlarged gallbladder were observed by prenatal ultrasound. Amniocentesis revealed the karyotype of the fetus as 46, XN, add(X) (p22.2) and the result of chromosomal microarray analysis was arr[hg19] Xq27.1q28(138,506,454–154896094) × 2 and arr[hg19] Xp22.33p22.32(168,551–5,616,964) × 1. CNV-seq showed that the mother shares a 16.42 Mb duplication in the Xq27.1-q28 region and a 2.97 Mb deletion in the Xp22.33-p22.32 region. After genetic counseling, the couple chose to terminate the pregnancy.

**Conclusion:**

The combination of NIPS and CMA would be of values in detection of subchromosomal duplications and/or deletions at fetal stage. The detection of X chromosome aberration in a male fetus should give suspicion of the possibility of maternal inheritance.

## Introduction

Chromosomal copy number variation (CNV) can lead to a variety of neonatal abnormalities, such as mental retardation and developmental delay. Although CNV is one of the important causes of birth defects, it is not easily detected by ultrasound in early pregnancy. With the application of Chromosomal microarray analysis (CMA) technology, genome-wide detection of CNVs becomes possible, which can identify CNVs with high resolution [1]. Furthermore, it has been evidenced that noninvasive prenatal screening (NIPS) can detect fetal chromosomal abnormalities including CNV from cell-free fetal DNA (cffDNA) in maternal peripheral blood as early as 13 weeks gestation period [2].

It has been recognized that patients with a total or partial deletion of the short arm of the X chromosome usually have the characterization of short stature and may carry variable features of Turner syndrome (TS) [3]. Deletion of the *SHXO* gene located in Xp22.32 region was known to be associated with short stature as well as some additional stigmata of TS, and its insufficient haploid dosage would lead to Leri-Weill dyschondrosteosis [4].

The frequency of Xq chromosome duplications is rare, and majority of Xq duplications observed in males are inherited from phenotypically normal or near-normal mothers [5]. Duplications of Xqter containing the *MECP2* gene are frequently detected. The duplication of Xq26-q28 chromosome region yields recognizable phenotypes, including distinctive facial features, major axial hypotonia, severe developmental delays, severe feeding difficulties, abnormal genitalia, and susceptibility to infection [6–9]. The critical dosage-sensitive *MECP2* gene, located at Xq28, is the main gene responsible for these severe phenotypes.

In this case report, we identify a male fetus with suspected maternal inheritance of Xp22.33p22.3 deletion and Xq27.1q28 duplication by the prenatal analysis. Moreover, we compared the similarities between prenatal ultrasound findings from the fetus and the clinical features described in the literature in carriers with similar Xp22.33p22.3 deletion and Xp27.1q28 duplication.

### Case report

**Fig. 1:**
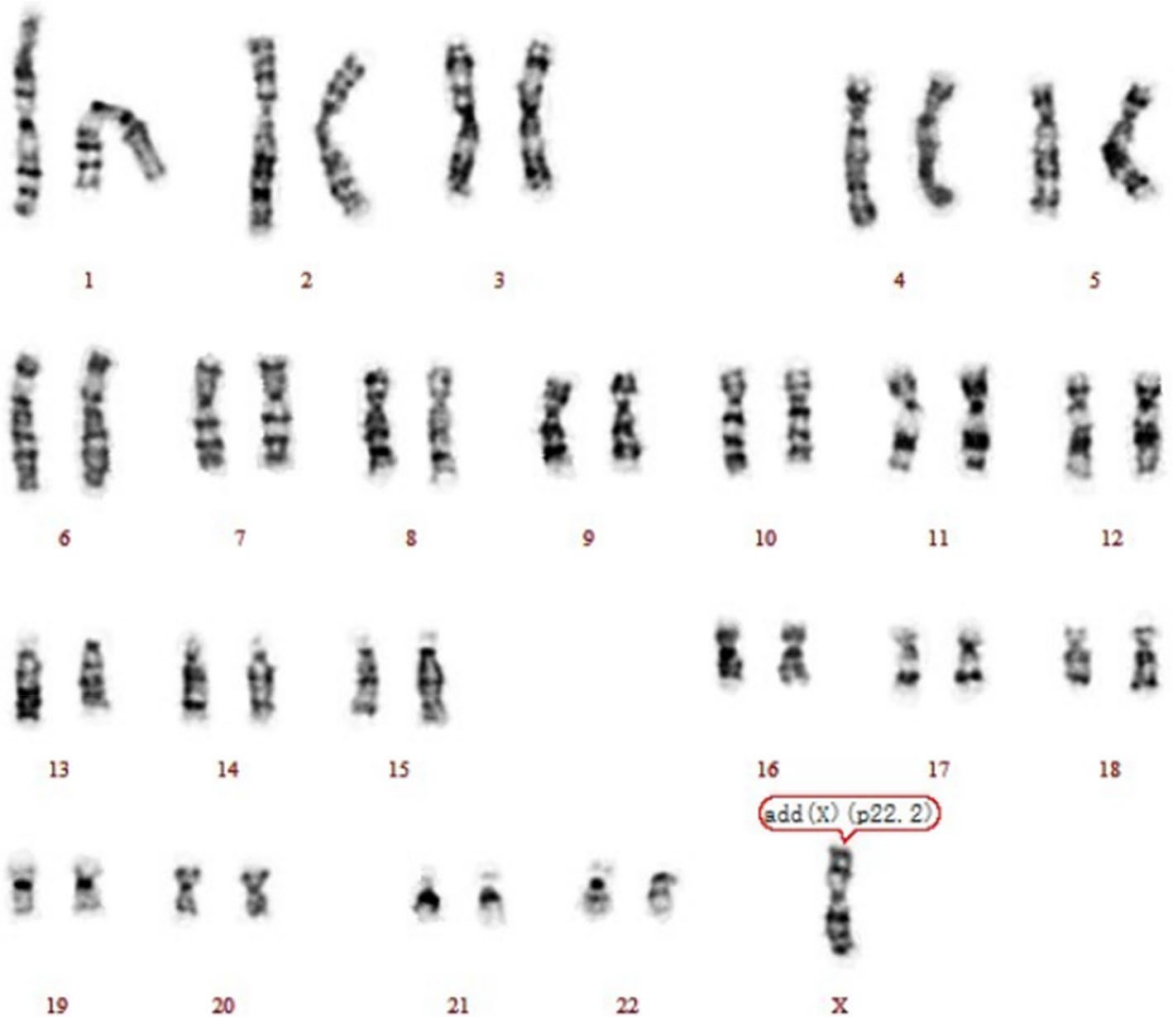
A karyotype of 46, XN, add(X) (p22.2) in the fetus

**Fig. 2:**
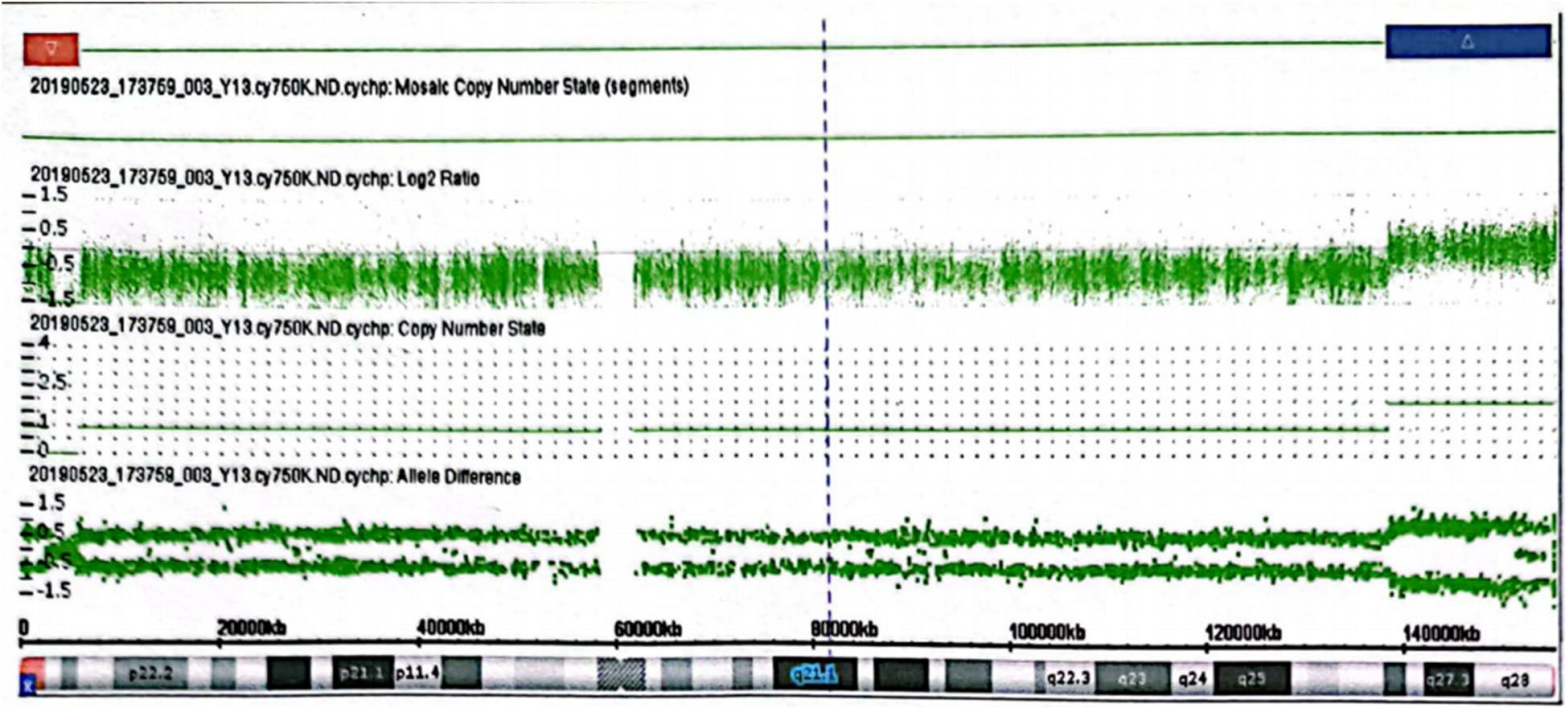
The result of CMA revealed a 5.40 Mb deletion (red arrow) in Xp22.33p22.32 and a 16.4 Mb duplication (blue arrow) in Xq27.1q28

**Fig. 3:**
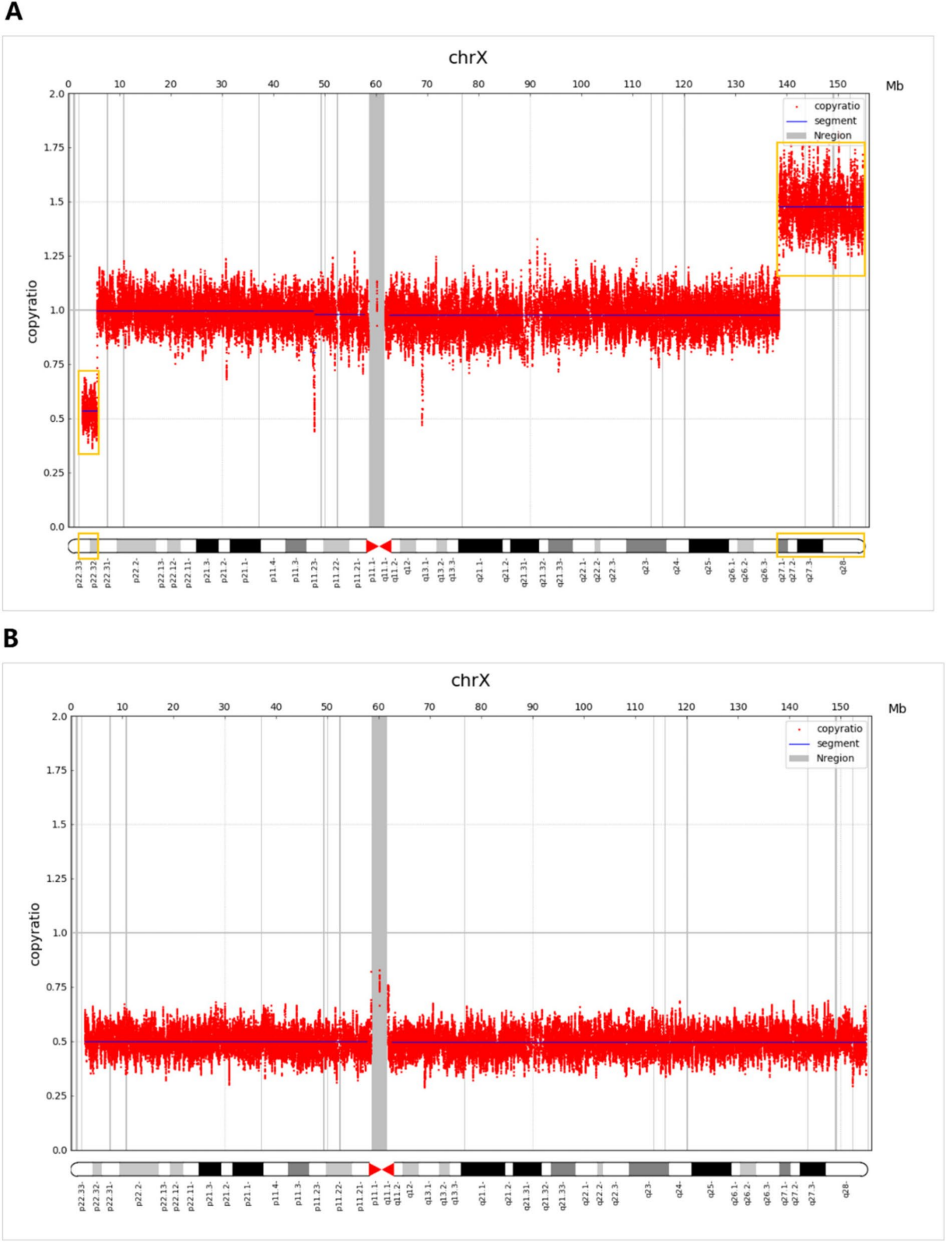
A 2.97 Mb deletion on Xp22.33p22.32 and a 16.42 Mb duplication on Xq27.1q28 were identified in the mother (**A**) and no significant abnormality was found on the father's X chromosome (**B**)

A 36-year-old, gravida 7, para 2, woman was referred to the prenatal diagnosis center of our hospital because her NIPS result indicated the presence of abnormalities in X chromosome, namely del (Xp22.33-p22.32, 2.28 M) and dup (Xp27.1-q28, 9.07 M). The NIPS was performed at the 17^+3^ weeks of gestation, and samples were sequenced on the NextSeq CN500 platform (Berry Genomics, Beijing, China). Her body height is 150 cm and body weight is 48.6 kg. She had experienced five miscarriages, including one induced abortion and four spontaneous abortions. Briefly, in March 2007, the male embryo stopped developing; in April 2009, she took drugs to induce labor during pregnancy; in 2013, the embryo was aborted at 50 days of gestation; in April 2014, a male fetus was detected with lobar holoprosencephaly; In 2015, the embryo was aborted at 60 days of gestation. The couple are non-consanguineous and have no history of exposure to toxic and harmful substances. Routine ultrasound examination showed multiple congenital malformations, hydrocephalus and gallbladder enlargement. Routine chromosome analysis by G-banding techniques at 320 bands of resolution was performed and the fetus karyotype was described as 46, XN, add(X) (p22.2), showing chromosome fragments of unknown origin were attached to Xp22.2 (Fig. 1). No abnormality was revealed in the result of QF-PCR. CMA was performed on amniotic fluid cells according to the manufacturer's protocol by CytoScan 750 K array (Affymetrix, Santa Clara, CA). The result revealed a 16.4 Mb duplication of Xq27.1-q28 and a 5.4 Mb deletion of Xp22.33-p22.32 region, shown as arr[hg19] Xq27.1-q28 (138,506,454–154896094) × 2, arr[hg19] Xp22.33-p22.32 (168,551–5,616,964) × 1 (Fig. 2). Copy number variation sequencing (CNV-seq) was carried out for parents. In the presence of a normal X chromosome, the mother but not the father was found to harbor similar 2.97 Mb microdeletion of the Xp22.33-p22.32 region and 16.42 Mb duplication of the Xq27.1-q28 region (Fig. 3). After comprehensive multidisciplinary counseling, the couple opted to terminate the pregnancy.

## Discussion

In this report, we described an uncommon prenatal diagnosis case, which carried two abnormalities on X chromosome, including an Xp22.33-p22.32 deletion and an Xq27.1-q28 duplication. The literature currently contains only a few prenatal case reports involving such Xq duplications and Xp deletions.

Duplication of the terminal long arm segment Xq27.1-qter was uncommon, and interstitial duplications encompassing the Xq27.1-Xq28 region have only been reported in a small number of patients [10]. In the case reported here, the Xq27.1-q28 duplication was detected in both the maternal and the fetal chromosome. And of note, the mother had a normal phenotype. Studies have shown that duplications located on the X chromosome were always prone to have more severe effects on males, as these imbalances could result in functional disomy of genes located within the duplicated segment. In contrast, because of the inactivation of one skewed X chromosome, female carriers were usually asymptomatic or mildly abnormal [8, 11]. To better generalize the clinical features related to Xq27.1-Xq28 duplication, the clinical data of the patients with the similar duplication as that of this case are summarized in Table 1 [12, 13, 14, 15, 16, 17]. The patients showed some consistent phenotypes: dysmorphic features (8/14), small testes (8/14), hands or feet abnormalities (7/14), short stature (6/14), developmental retardation (5/14), intellectual disability (5/14), undescended testis (5/14), myelomeningocele (5/14), Intrauterine Growth Retardation (IUGR) (3/14). In addition, some patients suffer from symptoms of absent speech, obesity, megalothymus, acute sex, as well as low levels of luteinizing hormone, follicle stimulating hormone, testosterone, and GH deficiency.

**Table 1:** Clinical features of subjects with duplications involved in chromosome Xq27.1q28

**Reference**	**Rio et al. **[12]			**Hickey et al. **[13]	**Hureaux et al. **[14]			**Zhuang et al. **[15]	**Wei et al. **[16]	**Arya et al. **[17]					
	**Case 1**	**Case 2**	**Case 3**	**Case 1**	**Case 1**	**Case 2**	**Case 3**	**Case 1**	**Case 1**	**Case 1**	**Case 2**	**Case 3**	**Case 4**	**Case 5**	**Our case**
**Age**	35 years	59 years	29 years	36 years	Fetus	Fetus	Fetus	7 years	5 years	NA	2.32 years	Fetus	NA	NA	Prenatal
**Size/location**	5.1 Mb/Xq27.3q28	5.1 Mb/Xq27.3q28	5.1 Mb/Xq27.3q28	Xq27.3q28	662 kb/Xq27.1	560 kb/Xq27.1	9.1 Mb/Xq27.1q28	2.2 Mb/Xq27.1q27.2	1.4 Mb/Xq27.1q27.2	323.8 kb/Xq27.1	396 kb/Xq27.1	481 kb/Xq27.1	11 Mb/Xq27.1	481 kb/Xq27.1	16.4Mb/Xq27.1q28
**IUGR**	+	+	+	NA	NA	NA	NA	NA	NA	NA	NA	NA	NA	NA	Hydrocephalus; multiple congenital malformations; enlarged gallbladder
**Developmental retardation**	NA	NA	NA	+	NA	NA	NA	NA	NA	+	NA	+	+	+	
**Absent speech**	-	-	-	+	NA	NA	NA	NA	NA	NA	NA	NA	NA	NA	
**Intellectual disability**	+	+	+	+	NA	NA	NA	NA	-	NA	NA	NA	NA	+	
**Short stature**	+	+	+	+	NA	NA	NA	NA	-	NA	NA	NA	+	+	
**Hands or feet abnormalities**	+	+	+	+	+	+	+	NA	-	NA	NA	NA	NA	NA	
**Dysmorphic features**	+	+	+	+	+	+	+	-	-	NA	+	NA	NA	NA	
**Undescended testis**	+	+	+	+	NA	NA	NA	-	+	NA	NA	NA	NA	NA	
**Small testes**	+	+	+	+	NA	NA	NA	-	-	+	+	+	NA	+	
**Obesity**	NA	NA	NA	+	NA	NA	NA	-	NA	NA	NA	NA	NA	NA	
**Myelomeningocele**	-	-	-	-	+	+	+	NA	NA	NA	+	+	NA	NA	
**Chiari II malformation**	-	-	-	-	+	+	+	NA	NA	NA	NA	NA	NA	NA	
**Megalothymus**	NA	NA	NA	NA	NA	NA	+	NA	NA	NA	NA	NA	NA	NA	
**Ambiguous sex**	NA	NA	NA	NA	NA	NA	NA	+	NA	NA	NA	NA	NA	NA	
**MRI detection**	NA	NA	NA	NA	NA	NA	NA	NA	NA	Brain MRI showed a hypoplastic anterior pituitary and ectopic posterior pituitary tissue bright signal (T1)	MRI identified hydrocephalus and agenesis of the corpus callosum	Pituitary MRI and the initial endocrine evaluation were normal	Pituitary MRI showed a hypoplastic anterior pituitary and ectopic posterior pituitary bright signal	Brain MRI showed partial agenesis of the corpus callosum, an absent septum pellucidum and the presence of heterotopic grey matter	
**Endocrine investigation**	NA	NA	NA	Low serum testosterone; low normal FSH and LH; low IGF-1; comprehensive metabolic profile and thyroid function tests were normal.	NA	NA	NA	Low luteinizing hormone, follicle-stimulating hormone, testosterone; serum progesterone and prolactin were normal.	Low luteinizing hormone; normal follicle stimulating hormone, anti-mullerian hormone, prolactin, and 17-α-hydroxyprogesterone.	Adrenal insufficiency and central hypothyroidism	GH deficiency	Normal	GH deficiency and borderline TSH deficiency.	GH deficiency	

*NA* Not available

In our case, the fetal was found to have multiple congenital malformations, hydrocephalus, and gallbladder enlargement by ultrasound. Fu et al. [18] suggested these clinical features such as hydrocephalus, ventriculomegaly, agenesis of the corpus callosum, choroid plexus cysts, intrauterine growth restriction, and hydronephrosis may be common sonographic features in fetuses with *MECP2* duplication syndrome (MDS). Notably, prenatal cases containing the Xq27.1-q28 duplication and Xp22.33-p22.32 deletion have rarely been reported, which limits our exploration of the correlation between the abnormal ultrasound diagnosis and the presence of X chromosome aberrations. Sun et al. [10] reported a prenatal case with 2q13 deletion and Xq27.1-q28 duplication, whose ultrasound examination only showed nasal bone dysplasia, without gallbladder enlargement and congenital multiple malformations as found in the present case. However, some studies [19–22] demonstrated that gallbladder enlargement is a high-risk indicator of aneuploid chromosomal abnormalities or biliary abnormalities. Sepulveda et al. [23] reported eight prenatal cases of gallbladder enlargement, of which four cases were also found to have other malformations. Chromosome examination was performed in these 4 cases, and aneuploid chromosome abnormalities were found in 3 of them.

**Fig. 4:**
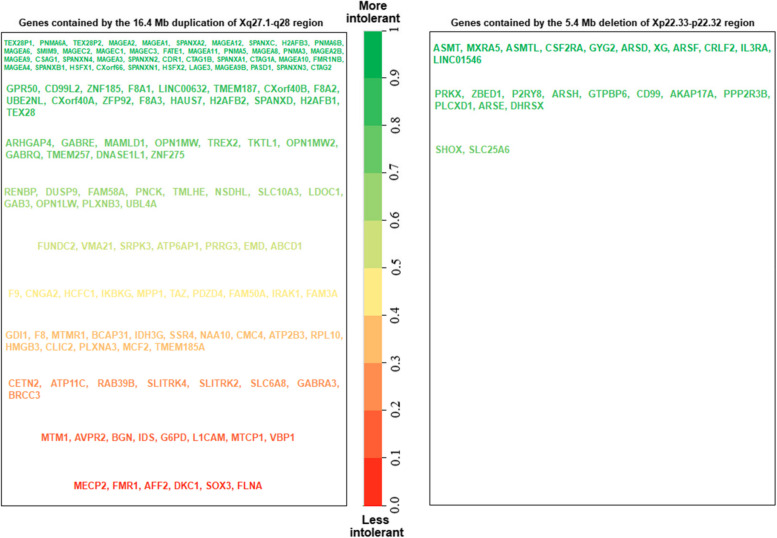
The genes within the Xq27.1-q28 region and Xp22.33-p22.32 region were marked with different colors, depending on their intolerance to mutations. Known pathogenic genes are marked in green (according to DECIPHER v11.23 database)

To explore possible diseases caused by the chromosomal aberrations in current case, we performed an analysis of the involved genes and their pathogenicity based on the Online Mendelian Inheritance in Man (OMIM) database. The results revealed 16.4 Mb duplication of Xq27.1-q28 region encompassed the 137 protein-coding genes (Fig. 4), including Methyl CpG binding protein 2 (*MECP2*). *MECP2* is located at Xq28 and is a key dose-sensitive gene. Its deletion or loss-of-function mutation could be the cause of progressive neurological disorder Rett syndrome, while the duplication or gain-of mutation of this gene can lead to MDS. *MECP2* and *IRAK1* genes constitute the minimal repeat region of MDS repeats, with reported repeats ranging from 0.079 to 15.8 Mb [24, 25]. Shao et al. [26] analyzed 5380 male cases and found that the duplication of Xq28 including MECP2 was the most common duplication in their cohort study. Duplications can be maternally inherited and the location and gene content are mainly maintained. Lubs et al. described a family of five affected boys with an Xq28 duplication inherited from carrier mothers which later were confirmed to be proper cases of MDS [27, 28]. Moreover, Yi et al. addressed that the transmission of the duplication is not always stable and its size may increase or decrease when transmitted from mothers to children [29].

Of the genes contained in this duplicated region, several are of particular interest. *FMR1* (OMIM 309550) encodes the Fragile X mental retardation protein, which plays an important role in the early stage of development and throughout life. Inactivation of the *FMR1* gene results in fragile X mental retardation syndrome, while a duplication of *FMR1* has been reported to be related with characterized such as short stature, hypogonadism and facial dysmorphism [12, 13]. *SOX3* (OMIM 313430) is a single exon gene located in Xq27.1, which plays a key role in regulating embryogenesis and central nervous system development [30]. Over-expression or under-expression of *SOX3* can lead to similar clinical manifestations, including isolated GH defciency, congenital hypopituitarism [31], with or without intellectual impairment [32, 33]. Arya et al. [17] reported five men diagnosed with congenital pituitary dysfunction and presented with micropenis or cryptorchidism, pituitary structural abnormalities or other cranial midline lesions, such as dysplasia of corpus callosum and absence of pellucidum, all of whom were found to have Xq27.1 duplications including *SOX3*. In addition, XX male sex reversal has been reported to be associated with deletion and duplication in the upstream region of *SOX3* [34]. *FLNA* (OMIM 300049) gene is located on Xq28 and encodes filamin A protein. Deletion or duplication of the *FLNA* gene can affect close genes on the X chromosome, leading to a number of other signs and symptoms, such as neurological abnormalities and intellectual disability [35]. *AFF2* (OMIM 300806) is located in Xq28, which is highly expressed in a region of the human brain associated with learning, cognition, and memory. Whibley et al. [36] reported on a boy with mild intellectual disability who carried 210 kb of microduplications of the *AFF2* gene, and Rocha et al. [37] reported a case of partial *AFF2* microduplication with auditory processing disorders, emotional disorders, and macrosomia. These two cases suggest that partial *AFF2* duplication may be in association with normal IQ and behavioral problems.

In addition, a 5.4 Mb deletion was also detected in the Xp22.33-p22.32 region. This deletion covered Pseudoautosomal Region 1 (PAR1), which involves 23 protein-coding genes (Fig. 4), including *SHOX*. The *SHOX* gene, located at the very tip of the short arms of both sex chromosomes, encodes short stature homeobox protein. Loss of function of the *SHOX* gene in the pseudoautosomal region of Xp (haploidy insufficiency) may contribute to short stature and skeletal characteristics in patients [38], and was associated with Leri-Weill dyschondrosteosis, Langer mesomelic dysplasia, and X-linked idiopathic familial short stature [39]. To further explore the genotypic-phenotypic association, we summarized the clinical data of Xp22.33-p22.32 deletion similar to this case [40–44]. As shown in Table 2, Xp22.33-p22.32 deletion may lead to multiple developmental defects such as short stature (5/6), inheritance/ family history of short stature (4/6), subtly short 4th and 5th metacarpal bones (3/6) and slightly delayed bone ages (3/6). In some cases, there were other abnormal findings such as facial dysmorphism, low-set ears and short limbs.

**Table 2:** Clinical features of subjects with deletions involved in chromosome Xp22.33p22.32

**Reference**	**Chen et al. **[40]	**Chen et al. **[41]	**Cho et al. **[42]		**Schwinger et al. **[43]	**D’Ambrosio et al. **[44]	
	**case 1**	**case 2**	**case 3**	**case 4**	**case 5**	**case 6**	**our case**
**Gender/age**	female/prenatal	female/prenatal	female/8 years, 9 months	female/11 years, 10 months	female/10 years old	female/11 year, 9 months	fetus/prenatal
**Size/location**	4.56 Mb/Xp22.33p22.32	213.9 kb/Xp22.33	Xp22.33-Xp22.12	Xp22.33-Xp22.12	Xp22.33-Xp22.32	21.26 Mb/Xp 22.33p22.12	5.4 Mb/Xp22.33p22.32
**Height**	23.5 cm	49.5 cm	118.6 cm	137.9 cm	127.5 cm	136.5 cm	NA
**Short stature**	+	-	+	+	+	+	NA
**Inheritance/Family history of short stature**	NA	+	+	+	+	-	NA
**Dysmorphic features**	+	-	-	-	-	NA	NA
**Maternal height**	147 cm	150 cm	148.7 cm	148.7 cm	150 cm	143.8 cm	150 cm
**Paternal height**	170 cm	170 cm	170 cm	170 cm	NA	165 cm	NA
**Karyotype**	46,Y,der(X)t(X;?)(p22.31;?)	46,XY	46,X,del(X)(p22.1)	46,X,del(X)(p21.3)	46,X,del(X)(p22.3)	46, X,del(X)p	46, XN, add(X) (p22.2)
**Other**	Prenatal ultrasound at 20 weeks of gestation revealed a hypoplastic left heart; low-set ears; short limbs		Relatively small hands; subtly short 4th and 5th metacarpal bones; slightly delayed bone ages	Relatively small hands; subtly short 4th and 5th metacarpal bones; slightly delayed bone ages		Foreshortening of the 4th and borderline length of the 5th metacarpal bones with positive metacarpal sign for TS; slightly delayed bone ages; abnormally large and deformed ears with underdeveloped auricles; overlapped and malformed teeth; low posterior hairline; mildly flexible joints; pes planus; breast and genitalia exam were compatible with Tanner stage III	Prenatal ultrasound showed multiple congenital malformations, hydrocephalus, and enlarged gallbladder

*NA* Not available

As the technological advances of high-throughput sequencing and bioinformatics analysis, NIPS is no longer limited to screen fetal chromosome aneuploidy, but is gradually extended to the field of CNV detection. As reported in this study, NIPS technology was successfully used to predict the deletion and duplication on the X chromosome, and the results were consistent with the detection of CMA. In this case, karyotype analysis revealed that a chromosome fragment of unknown origin attached to Xp22.2 and and QF-PCR failed to detect the subchromosomal deletion and duplication. It is therefore suggested that the traditional G-banding chromosome karyotype analysis and QF-PCR have limitations in the accurate detection of chromosome deletion and duplication at the submicroscopic level [45]. Compared with conventional cytogenetic methods, CMA technology using millions of probes and provides better resolution, is a more effective means of assessing ploidy for specific chromosomes as well as the specific location of chromosomal abnormalitie by detecting the microdeletions and microduplications in chromosomes. Studies have shown that for the regions of CNVs > 5 Mb; the clinical sensitivity of CMA analysis can reach 90.9% with a clinical specificity of 95%, while for the regions of CNVs < 5 Mb, the clinical sensitivity may drop to 14.3% with a clinical specificity of 100% [46]. When comparing the efficacy of NIPT, karyotype analysis and CMA in CNVs detection, CMA is still the most effective method [47].

It is worth noting that although NIPT has shown great potential in detecting fetal CNVs, there are still false positive and false negative results [48]. Its detection results will be affected by the variation size, fetal fraction, sequencing depth, and biological variability of CNVs (GC bias, repeating elements) [49]. At present, due to the lack of clinical efficacy data for detecting CNVs, non-invasive detection techniques that are widely used for prenatal detection of CNVs require more clinical validation research before they can be put into clinical practice [50]. Therefore, detailed genetic counseling should be conducted before implementing NIPS. NIPS is not recommended for routine screening for CNVs with cffDNA [51] or screening for genome-wide CNVs. When pathogenic CNVs are identified by NIPS, patients should be referred to an experienced geneticist, and invasive diagnostic confirmation options should be provided [52]. It can be found that prenatal diagnosis is a field that involves multidisciplinary cooperation, such as obstetrics, imaging, genetics, statistics and research. Interdisciplinary cooperation plays a vital role in improving the accuracy of fetal malformation diagnosis.

In summary, taken together the combination of NIPS and prenatal diagnostic methods such as CMA technology effectively detect micro-duplication/micro-deletion on fetal chromosomes, providing more precise clinical diagnosis and genetic counseling for pregnant women and their families.

## Conclusion

In summary, a rare prenatal case with 5.4 Mb Xp22.33p22.32 deletion and 16.4 Mb Xq27.1q28 duplication is reported here. The relationship between these chromosomal structural abnormalities and clinical phenotypes in conjunction with other cases is discussed, aiming to provide more information about pathogenic CNVs. We suggest that detection of X chromosome aberration in a male fetus should give suspicion of the possibility of maternal inheritance. Of note, the development of NIPS and CMA plays an important role in detection of chromosome deletion and duplication at the submicroscopic level. Importantly, reports and studies of prenatally diagnosed cases help couples be in an informed way what the course of the pregnancy will be, so that they can better foresee and control the future.

## Data Availability

The datasets analyzed during the current study are available from the corresponding author on reasonable request.

## Abbreviations

NIPS: Noninvasive prenatal screening
CNV: Chromosomal copy number variation
cffDNA: Cell-free fetal DNA
TS: Turner syndrome
CNV-seq: Copy number variation sequencing
MDS: *MECP2* Duplication syndrome
MECP2: Methyl CpG binding protein 2
PAR1: Pseudoautosomal Region 1
